# The role of sex hormones in aldosterone biosynthesis and their potential impact on its mineralocorticoid receptor

**DOI:** 10.1097/XCE.0000000000000305

**Published:** 2024-06-05

**Authors:** Andrea Vecchiola, Thomas Uslar, Isidora Friedrich, Joaquin Aguirre, Alejandra Sandoval, Cristian A. Carvajal, Alejandra Tapia-Castillo, Alejandra Martínez-García, Carlos E. Fardella

**Affiliations:** a Departamento de Endocrinología, Facultad de Medicina, Centro Traslacional de Endocrinología UC (CETREN), Pontificia Universidad Católica de Chile; b Departamento de Endocrinologìa, Escuela de Medicina, Pontificia Universidad Católica de Chile, Santiago; c Escuela de Tecnología Médica, Facultad de Ciencias, Pontificia Universidad Católica de Valparaíso, Valparaíso, Chile

**Keywords:** aldosterone aldosterone biosynthesis, CYP11B2, mineralocorticoid receptor, sexual steroids

## Abstract

Blood pressure (BP) regulation is a complex process involving various hormones, including aldosterone and its mineralocorticoid receptor. Mineralocorticoid receptor is expressed in several tissues, including the kidney, and plays a crucial role in regulating BP by controlling the sodium and water balance. During different stages of life, hormonal changes can affect mineralocorticoid receptor activity and aldosterone levels, leading to changes in BP. Increasing evidence suggests that sex steroids modulate aldosterone levels. Estrogens, particularly estradiol, mediate aldosterone biosynthesis by activating classical estrogen receptors and the G protein-coupled receptor. Progesterone acts as an anti-mineralocorticoid by inhibiting the binding of aldosterone to the mineralocorticoid receptor. Moreover, progesterone inhibits aldosterone synthase enzymes. The effect of testosterone on aldosterone synthesis is still a subject of debate. However, certain studies show that testosterone downregulates the mRNA levels of aldosterone synthase, leading to decreased plasma aldosterone levels.

## Introduction

Aldosterone is the primary mineralocorticoid steroid hormone produced by the zona glomerulosa in response to high potassium, angiotensin II, and adrenocorticotropin stimulation [1,2]. It is a pivotal component of the renin-angiotensin-aldosterone system (RAAS), which plays a central role in the homeostatic regulation of blood pressure (BP); additionally, it stimulates sodium reabsorption and potassium excretion in the distal portion of the nephron [3–5].

Aldosterone is derived from cholesterol, which serves as the shared precursor for all steroid hormones, and is stored within intracellular lipid droplets and mobilized to the mitochondria upon stimulation. In the mitochondria, a series of enzymatic reactions involving cholesterol culminates in aldosterone production. The intramitochondrial cholesterol transfer is facilitated by the 30-kDa steroidogenic acute regulatory protein. Upon entering the mitochondria, cholesterol is converted to pregnenolone, a process activated by cytochrome P450 side-chain cleavage (CYP11A1) [6–8]. Following several subsequent enzymatic steps, it is ultimately transformed into aldosterone through the aldosterone synthase enzyme encoded by the *CYP11B2* gene [6,8].

Aldosterone readily crosses the cell membrane and attaches to mineralocorticoid receptors located within the cytoplasm. Upon binding aldosterone, the mineralocorticoid receptor dimerizes, translocates to the nucleus, and acts as a ligand-activated transcription factor, where it binds to specific regions of DNA containing mineralocorticoid receptor response elements [9]. The binding of the aldosterone–mineralocorticoid receptor complex to these DNA-responsive elements leads to transcription and translation of downstream genes. Notably, this process induces the expression of genes like serum- and glucocorticoid-stimulated kinase 1 (SGK1), which plays a pivotal role in the regulation and activation of ionic transport proteins, including the epithelial cell sodium channel (ENaC) in principal cells [10]. Subsequently, the activated ENaC promotes sodium and water reabsorption and regulates fluid volume and sodium balance [11]. Sodium homeostasis is regulated by proton pumps, such as H^+^-ATPase and H^+^-K^+^-ATPase, in intercalated cells [12,13]. Apart from the genomic pathway, aldosterone exerts a rapid impact through nongenomic pathways. In the cytoplasm, the aldosterone-mineralocorticoid receptor complex activates SGK1, which phosphorylates Nedd4-2 (neural precursor cell-expressed, developmentally downregulated protein 4-2), and induces its interaction with members of the 14-3-3 family of regulatory proteins. The concerted action of SGK1 and 14-3-3 appears to disrupt Nedd4-2-mediated ubiquitination of ENaC, thus providing a mechanism through which SGK1 modulates Na(+) current mediated by ENaC [14].

Increasing clinical and molecular evidence suggests that sex steroids modulate aldosterone levels and mineralocorticoid receptor expression and activity. Thus, during different stages of life, hormonal changes can affect the activity of mineralocorticoid receptor and aldosterone, aggravating the pathological effects of mineralocorticoid receptor on epithelial and endothelial targets. This in turn leads to changes in BP and increases the risk of cardiovascular and cerebrovascular events and renal damage.

## Clinical evidence of intermodulation between sex hormones and aldosterone on the mineralocorticoid receptor

Healthy women have higher levels of serum aldosterone in comparison to healthy men [15] with aldosterone levels increasing in the luteal phase of the menstrual cycle compared to the follicular phase. The elevation of progesterone levels during the luteal phase of the oestrous cycle and pregnancy leads to a compensatory increase in RAAS activity and aldosterone synthesis. This response is triggered by the decreased activity of mineralocorticoid receptor in aldosterone target cells, serving to the maintenance of homeostasis.

Moreover, sex hormones and age regulate aldosterone levels and play pivotal roles in the pathophysiology of hypertension in postmenopausal women [16]. The cessation of estrogen production in postmenopausal women leads to an increase in BP, and early estrogen replacement therapy could prevent this increase in BP and atherosclerotic progression [17,18]. Experimental studies have shown that circulating aldosterone levels increase after menopause [19], and estrogen therapy reduces this effect [20–26].

In contrast, the use of combined oral contraception increases aldosterone levels but decreases renin levels. It is hypothesized that oral contraception may contribute to hypertension through a ‘first-pass’ effect, where oral estrogens induce hepatic production of the renin substrate, angiotensinogen, leading to an increase in angiotensin I which is in turn converted to angiotensin II [27]. In a recent study, approximately 500 women aged 17–27 years were evaluated. Oral contraception users exhibited a 42% higher median aldosterone renin ratio (ARR) at 17 years of age and a 23% higher median ARR at 27 years in comparison to nonusers [28]. As expected, these changes may have led to false-positive primary aldosteronism screening results. In contrast to renin concentration, plasma renin activity remained unchanged during oral contraception treatment. This stability can be attributed to the compensatory effect of the elevated renin substrate, which is counteracted by the suppression of the renal release of active renin, as evidenced by a lower renin concentration [27]

In women using progesterone-only pills, the pronounced anti-mineralocorticoid receptor effect is predominantly associated with drospirenone, a progestin derived from spironolactone. However, this effect diminishes as drospirenone was removed from MRA due to its progestational activity. Nevertheless, this effect is not typically observed when progesterone-only pills containing levonorgestrel or desogestrel are used [29].

During pregnancy, aldosterone levels increase approximately tenfold. However, the pathophysiological effects of increased aldosterone on mineralocorticoid receptor are mitigated during pregnancy by progesterone, which acts as an antagonist of mineralocorticoid receptor and inhibits CYP11B2 activity in the adrenal tissue [30]. Plasma renin activity (PRA) can increase up to fourfold in pregnant women compared with normal women [31]. This increase is potentially due to a higher concentration of renin substrate and a twofold increase in active renin concentration [27].

In addition, in a study on pregnant women with familial hyperaldosteronism type I, a condition that occurs due to an unequal crossover of the genes encoding steroid 11ß-hydroxylase (CYP11B1) and aldosterone synthase (CYP11B2), resulting in a chimeric CYP11B1/CYP11B2 gene with aldosterone synthase activity regulated by plasmatic adrenocorticotropes, we observed an improvement in BP concomitant with the normalization of aldosterone and ARR during pregnancy. After delivery, while the progesterone and estradiol levels decreased, we observed an increase in aldosterone levels and suppression of PRA, leading to an anticipated rise in ARR [32]. This is intriguing due to the notable rise in adrenocorticotropic hormone concentration during pregnancy, which directly regulates aldosterone production in familial hyperaldosteronism type I. To date, the improvement in BP in familial hyperaldosteronism type I during pregnancy has been attributed to elevated levels of progesterone, acting as a competitive antagonist of the mineralocorticoid receptor.

Furthermore, in studies carried out by our group within a multigenerational family with familial hyperaldosteronism type I [33], spanning four generations, we noted a decline in aldosterone levels and ARR during the transition from childhood to adulthood. Thus, elevated aldosterone and ARR levels were observed exclusively in prepubertal children. In contrast, the adult population exhibited normal levels of aldosterone and PRA, and normal ARR. However, the presence of high levels of 18-hydroxy cortisol in both populations suggests that the 18-hydroxylation activity of the hybrid enzyme is maintained despite the normalization of aldosterone levels, suggesting a role of sex steroids in aldosterone regulation.

## Molecular evidence of estrogens in the biosynthesis of aldosterone

Estrogens are steroid hormones mainly synthesized in the ovaries. Estradiol (E2), one of the three forms of estrogen, has diverse biological functions and affects several tissues and organs. Estrogens exert a beneficial effect on BP regulation and cardiovascular disease risk by activating classical estrogen receptors (ERα and ERβ). Ligand-dependent estrogen signaling begins with the binding of estrogen to the estrogen receptor. In the absence of E2, these receptors bind to heat shock protein 90 and remain inactive. When activated, the cell-specific transcriptional response to estrogen depends on multiple factors, such as co-regulatory proteins and the characteristics of the promoters of estrogen-responsive genes. Since hormones are modulators of transcription, the pattern of modulated genes also depends on other signaling pathways that are active in the cell at the time of hormone exposure. A G protein-coupled receptor, GPER (also referred to as GPR30 and GPER-1), plays a role in mediating the rapid nongenomic effects of estrogen in various tissues and cells [34]. GPER was initially described as an estrogen-specific receptor but was subsequently discovered to promiscuously bind to other steroids and mediate mineralocorticoid receptor-independent aldosterone effects in different cell types [35–37].

Multiple studies have suggested a role for estrogens in the regulation of aldosterone under physiological and pathophysiological conditions. In the human fetal adrenal gland, ERβ expression was observed to be high, whereas ERα expression was observed to be low [38]. Notably, in the post-adrenarche, ERα was barely detectable in the adrenal gland, while ERβ was predominantly detected in the zona reticularis [39,40]. Additionally, more recently, the presence of GPER has been identified and found to be highly expressed not only in normal zona glomerulosa but also in aldosterone-producing adenoma (APA) tissue, where immunostaining was much stronger for GPER-1 than for ERβ [34,41].

Experimental in-vitro studies have demonstrated a dual estrogen effect that balances each other but has different manifestations depending on the specific rank of expression of estrogen receptors in the adrenal cortex. Initially, estrogens inhibit aldosterone synthesis by acting directly on ERβ [34]. However, when ERβ expression decreases or is antagonized, E2 potently stimulates aldosterone synthase expression and the release of aldosterone through a mechanism involving GPER-1 stimulation [42].

Under physiological conditions, estrogens inhibit aldosterone synthesis by acting on ERβ, which explains the non-induction of aldosterone secretion when administered *in vitro*. Whether estrogen-mediated aldosterone synthesis through ERβ is reconciled with the fact that healthy premenopausal women have higher serum aldosterone levels compared to healthy men of the same age through GPER activity, cannot be assured. It has been described, that in the adrenal glands, unlike the pituitary, GPER expression is not sexually dysmorphic [41,43].

**Fig. 1:**
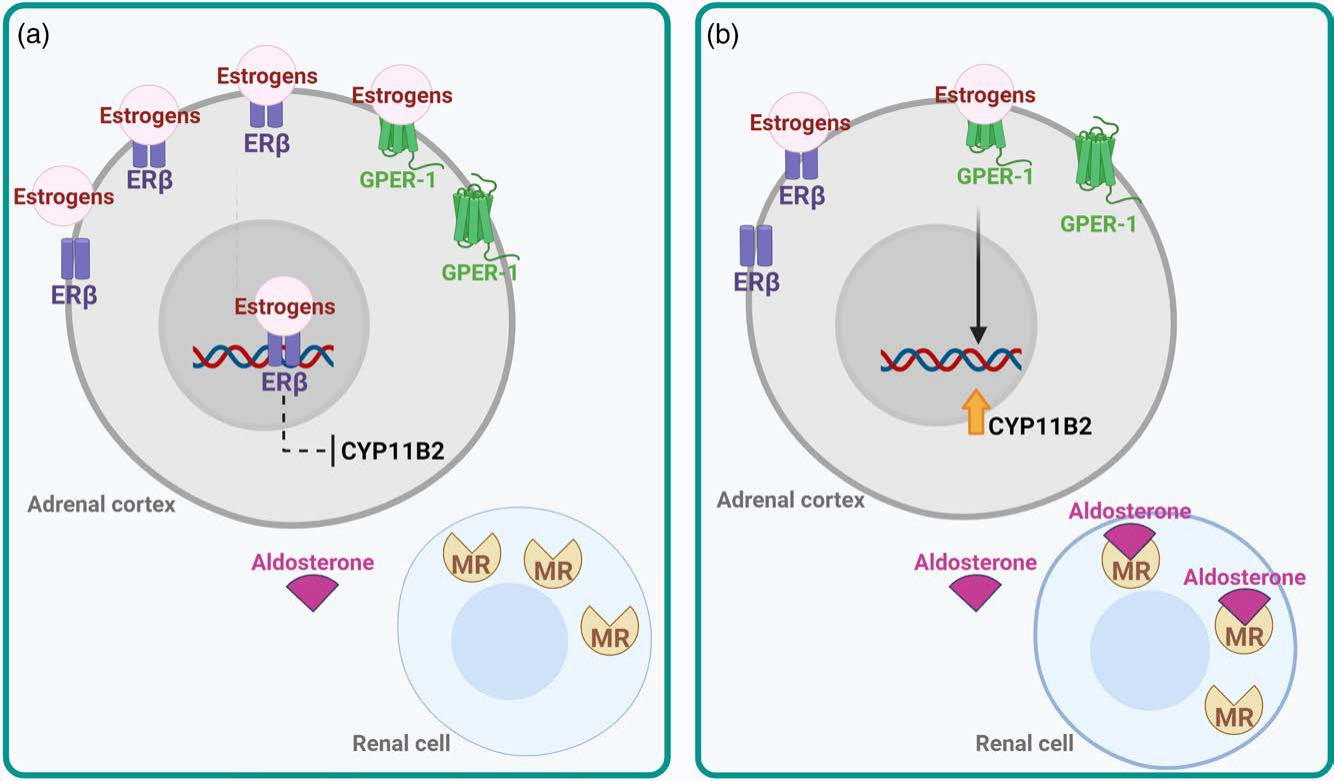
In premenopausal women (a), estrogens inhibit aldosterone synthesis by acting primarily on the ERβ. In menopausal women (b), cessation of estrogen production occurs, and estrogen induces aldosterone synthase through the activation of GPER. CYP11B2, aldosterone synthase, also called steroid 18-hydroxylase, corticosterone 18-monooxygenase or P450C18; ERβ, estrogen receptor beta; GPER, G protein-coupled estrogen receptor 1, also known as G protein-coupled receptor 30; MR, mineralocorticoid receptor.

In tissues without the tonic ERβ-inhibition such as APAs, estrogens may unmask a potent secretagogue effect on aldosterone via the GPER-1 receptor subtype [34]. Estradiol also regulates estrogen receptor expression. Thus, higher estrogen levels in premenopausal women and estrogen loss in postmenopausal women may affect the expression and relative amount of ERα, ERβ, and GPER in the adrenal cortex and then the aldosterone levels (Fig. 1).

It remains to be determined whether GPER density and its affinity for estradiol (E2) and related pathways change with age and postmenopausal status. However, since GPER is a G protein-coupled receptor activated by aldosterone itself and subject to GRK2-dependent desensitization [44], a process known to be affected by aging [45], it is plausible that aldosterone levels could be partially modulated by GRK2 in postmenopausal women.

Another likely regulatory mechanism is G protein signaling (RGS) proteins, which negatively regulate G protein-coupled receptors [46]. Certain RGS proteins, specifically RGS2 and RGS4, are known to regulate adrenal aldosterone synthesis in response to angiotensin II [47], and at least RGS2 has been reported to be suppressed by estrogen [48]. Given that AT_1_R-GPER interferes in the synthesis of aldosterone [49], it is quite plausible that RGS proteins are also involved in the effects of estrogens on adrenocortical aldosterone production.

## Molecular evidence of progesterone in the biosynthesis of aldosterone and its effect on mineralocorticoid receptor

**Fig. 2:**
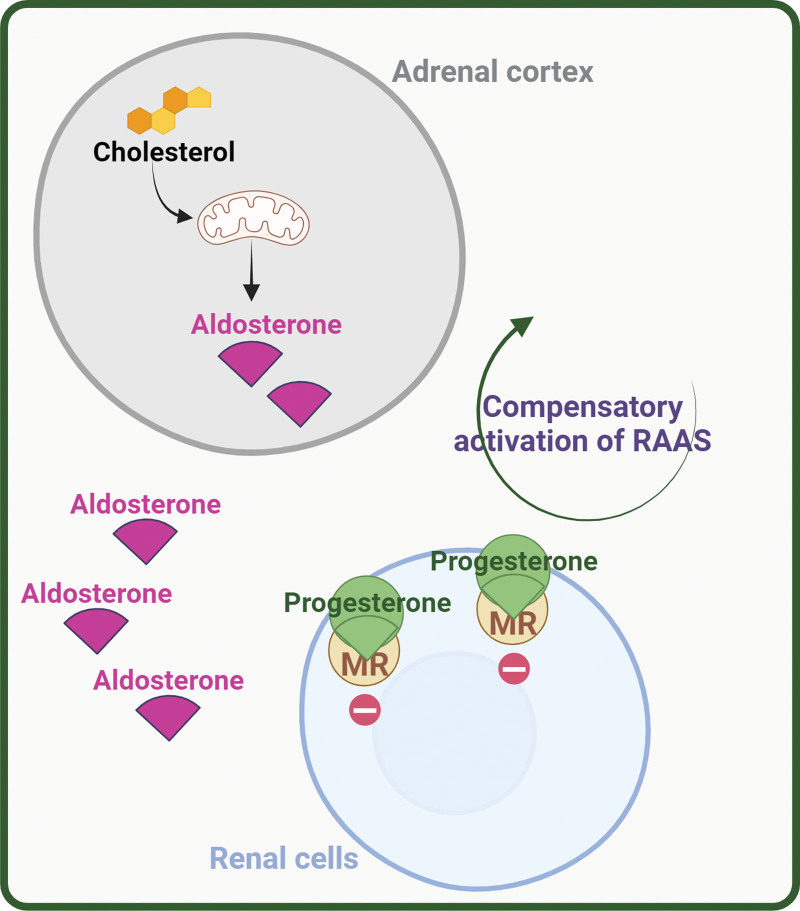
Progesterone, either by a physiological increase during the rat estrous cycle or induced, increases adrenal aldosterone production since it is the synthesis substrate. Progesterone could also increase blood aldosterone levels by inhibiting the binding of aldosterone to the mineralocorticoid receptor during the luteal phase of the menstrual cycle.

Progesterone is known to be an anti-mineralocorticoid compound that exerts a potent natriuretic effect primarily as a competitive inhibitor of aldosterone through the mineralocorticoid receptor in the distal renal tubule. This inhibition leads to heightened sodium excretion [50,51], a phenomenon observed in pregnant women or during the luteal phase of the menstrual cycle when aldosterone levels typically increase [52–54]. In pregnant women or the luteal phase of the menstrual cycle, progesterone levels increase by over 100- and 20-fold, respectively, and exhibit competitive anti-mineralocorticoid activity for mineralocorticoid receptor in nanomolar order (Ki 1.2 nM and IC50 11.0 nM) [55–57]. However, aldosterone can still act on mineralocorticoid receptor-responsive tissues due to the higher stability of the aldosterone-mineralocorticoid receptor complex in comparison to the progesterone-mineralocorticoid receptor complex. Furthermore, local inactivation of the progesterone pre-receptor by the enzyme 20-alpha-hydroxysteroid-dehydrogenase (AKR1C1), which converts progesterone to inactive 20-hydroxy-progesterone [58], exhibits reduced affinity for the human mineralocorticoid receptor (Ki 10.8 nM and IC50 282.5 nM) [51,59,60] (Fig. 2).

Progesterone may also directly influence adrenal aldosterone production. Using an in-vitro bioassay involving a human embryonic kidney cell line (HEK-293), we evaluated the effect of progesterone on the activity of wild-type human aldosterone synthase (CYP11B2) and a chimeric enzyme (CYP11B1/CYP11B2). Plasmids containing the wild-type and chimeric CYP11B1/CYP11B2 enzymes were transfected [61]. Progesterone competitively inhibited wild-type aldosterone synthase with similar efficacy and greater potency than the chimeric enzyme.

Finally, we observed that the hypothetical mechanism is related to progesterone as a substrate for aldosterone synthesis, and we could speculate that during physiologically high progesterone states, synthesis could be increased. Two human studies showed that the administration of oral micronized progesterone to healthy women for 4–8 days increased serum aldosterone levels but did not change PRA or plasma AngII [62,63]. In vitro, the addition of progesterone to isolated rat zona glomerulosa cells caused a 2.8-fold increase in aldosterone production, whereas the addition of estradiol had no effect. These data suggest that progesterone may have a direct influence on adrenal aldosterone production, indicating that this may be one of the mechanisms responsible for the increased aldosterone production observed in physiological states with high progesterone levels [64,65].

Thus, progesterone may exhibit distinct roles depending on its concentration and the physiological or experimental conditions. The following are the plausible roles played by progesterone: (1.) It is a mineralocorticoid receptor antagonist. (2.) It can potentially inhibit aldosterone synthase (wild-type and chimeric gene) enzymes. (3.) It is a precursor of aldosterone biosynthesis.

## Molecular evidence for the involvement of testosterone in the biosynthesis of aldosterone

The effects of testosterone on aldosterone levels showed discrepancies in cellular response parameters, suggesting that the effects of gonadal hormones on adrenocortical function vary according to species, sex, age, season, and environmental conditions.

Studies have shown that the androgen receptor is expressed in fetal adrenals [66]. Moreover, testosterone has been found to exert an inhibitory effect on basal aldosterone release [67], suggesting that androgens may have a direct effect on development and fetal adrenal gland function. In animal studies, prenatal exposure to testosterone has been shown to alter the adrenal expression of critical genes involved in steroidogenesis and hormone production. One of these studies by More *et al*. reported that in 6-month-old female rats prenatally exposed to testosterone, the levels of aldosterone synthase (CYP11B2) mRNA reduced, but this effect was not observed in other adrenal steroidogenic genes. Consequently, plasma aldosterone levels were lower in testosterone female rats [68]. Therefore, testosterone plausibly downregulates adrenal CYP11B2 mRNA levels, causing a decrease in plasma aldosterone levels.

Kau *et al*. [67] showed variation in aldosterone levels after castration in male rats. After orchidectomy, plasma aldosterone levels increased in male rats and were restored by testosterone replacement. Similar results were reported by Carsia *et al*. in orchidectomized lizard adrenocortical cells [69]. One study used Wistar rats and spontaneously hypertensive stroke-prone rats on a canola oil diet that significantly decreased testosterone levels in the testes and plasma. The decrease in plasma testosterone levels was accompanied by an increase in plasma aldosterone [70]. In bovine adrenal cells, testosterone hemisuccinate stimulates angiotensin membrane binding and aldosterone biosynthesis. In 2009, Yanes *et al*. [71] observed that male Dahl salt-sensitive rats on a low-salt diet demonstrated higher levels of intrarenal angiotensinogen mRNA in comparison to female rats. A high-salt diet for 4 weeks increased renal cortical angiotensinogen mRNA and protein levels only in male Dahl salt-sensitive rats, which was prevented by castration. Testosterone replacement in castrated Dahl salt-sensitive rats increased BP, renal injury, and the upregulation of renal angiotensinogen associated with the high-salt diet. The authors suggested that testosterone contributes to the development of hypertension and renal injury in male Dahl salt-sensitive rats fed a high-salt diet, possibly through the upregulation of the intrarenal renin-angiotensin system.

Testosterone and the synthetic androgen methylandrostenediol decrease the expression of CYP11B2 mRNA in the adrenal mitochondria of female rats [72]. Additionally, testosterone and two testosterone analogs inhibit human wild-type aldosterone synthase [73,74].

### Conclusion and perspectives

An expanding body of research suggests that multiple components within the RAAS pathway are modulated by sex hormones. Evidence has demonstrated that estrogen, progesterone, and testosterone use both common and distinct mechanisms to regulate aldosterone synthesis. Estrogen, for instance, exerts its influence directly through the ERβ receptor, thereby assuming a protective role in the regulation of BP and the mitigation of cardiovascular risk, particularly in premenopausal women. Conversely, the decline in estrogen production observed in postmenopausal women is associated with elevated BP and the advancement of atherosclerosis. Recent research has shed light on the impact of decreasing ERβ expression or distressing it and concomitant stimulation of aldosterone release through GPER-1 activation. This activation induces the expression of aldosterone synthase, potentially contributing to hypertension in postmenopausal women.

In contrast, both progesterone and testosterone inhibit aldosterone synthase. Nevertheless, progesterone counteracts the reduction in aldosterone synthesis by acting as an antagonist of the mineralocorticoid receptor, increasing aldosterone levels through the stimulation of the renin-angiotensin axis. Conversely, testosterone downregulates aldosterone synthase mRNA levels, resulting in a subsequent decrease in plasma aldosterone levels. This phenomenon has been observed in humans, where men consistently exhibit lower aldosterone levels than women who are specifically in the premenopausal state.

## Acknowledgements

This work was supported by Chilean grants from the Comisión Nacional de Investigación Científica y Tecnológica de Chile (CONICYT), FONDECYT 1160695 and 1212006, Centro Traslacional de Endocrinología UC (CETREN-UC), Pontificia Universidad Católica de Chile, Proyecto SOCHED 2022-6 Dra Ale Martínez García.

A.V. and C.E.F. designed the work; A.V., I.F., and J.A. contributed to the acquisition, collection, and review of papers; A.V., C.A.C., A.T.-C. contributed to the animals and biological review of data; C.E.F., A.M.-G., and T.U. contributed to the clinical review of data. All authors contributed to the revision and discussion of the manuscript. A.V., A.S., and A.T.-C. contributed to the figure design. All authors read and approved the final manuscript.

### Conflicts of interest

There are no conflicts of interest.
